# Restoring anisotropy after myocardial injury: strategies to align transplanted human induced pluripotent stem-cell-derived cardiomyocytes

**DOI:** 10.1038/s12276-026-01773-5

**Published:** 2026-07-03

**Authors:** Yura Son, Yong Yang, Mehdi Nikkhah, Wuqiang Zhu

**Affiliations:** 1 Department of Cardiovascular Diseases, Department of Physiology and Biomedical Engineering, and Center for Regenerative Medicine, Mayo Clinic Arizona, Phoenix, AZ 85054 USA; 2 Department of Biomedical Engineering, University of North Texas, Denton, TX 76207 USA; 3 School of Biological and Health Systems Engineering, Arizona State University, Tempe, AZ 85287 USA

**Keywords:** Heart stem cells, Induced pluripotent stem cells, Regeneration

## Abstract

Human induced pluripotent stem cell-derived cardiomyocytes (hiPSC-CMs) represent a promising cell source for cardiac regeneration, yet their therapeutic efficacy remains limited by poor structural organization and incomplete electromechanical integration after transplantation. A fundamental contribution to these limitations is made by the differences between the highly anisotropic architecture of native myocardium and the disorganized configuration of transplanted hiPSC-CMs. In the adult heart, cardiomyocyte alignment governs directional electrical conduction, coordinated contraction and efficient force transmission. In contrast, immature and poorly aligned hiPSC-CM grafts exhibit heterogeneous coupling, slowed or isotropic conduction and reduced functional integration. This review focuses on cardiomyocyte alignment as a critical design requirement for improving electrophysiological and regenerative outcomes in hiPSC-CM based cardiac cell therapy. We summarize bioengineering strategies that impose or preserve alignments, including topographical guidance, mechanical anisotropy, electrical conditioning and architectural delivery formats such as engineered tissues and cardiac patches. We further discuss how alignment influences intercellular junction organization, conduction anisotropy and graft–host integration and highlight the limitations of relying on global functional metrics that fail to capture directional electrical behaviour after transplantation. Collectively, this review argues that restoring myocardial anisotropy through engineered alignment is essential for translating hiPSC-CM immaturity towards functional and durable cardiac repair.

## Aligning Stem-Cell-Derived Cardiomyocytes for Better Heart Therapy

Human induced pluripotent stem cell-derived cardiomyocytes (hiPSC-CMs) offer a promising avenue for cardiac regeneration owing to their scalable production and inherent electrical and contractile properties. However, their integration into damaged heart tissue is hindered by poor cell retention and immature structural features. This review emphasizes the critical role of cardiomyocyte alignment in enhancing the functional integration of hiPSC-CMs. The authors explore bioengineering strategies that promote alignment, such as topographical guidance, mechanical conditioning and electrical stimulation, to improve structural organization and directional electrical conduction. Aligned hiPSC-CMs exhibit enhanced calcium transients and excitation–contraction coupling, which are crucial for effective cardiac repair. The review suggests that future research should focus on developing scalable delivery methods that maintain alignment post-implantation, ensuring continuity with host tissue.

This summary was initially drafted using artificial intelligence, then revised and fact-checked by the author.

## Introduction

Human induced pluripotent stem cell-derived cardiomyocytes (hiPSC-CMs) are a promising cell source for cardiac regeneration because they can be generated at scale and possess the intrinsic machinery required for electrical excitation and contraction^1–4^. Despite these advantages, the functional integration of transplanted hiPSC-CMs into injured myocardium remains limited, and durable improvements in cardiac performance are inconsistent across studies^5–7^. Poor cell retention, immature sarcomere organization and incomplete electromechanical behaviour continue to constrain therapeutic efficacy and raise safety concerns^8^.

**Fig. 1:**
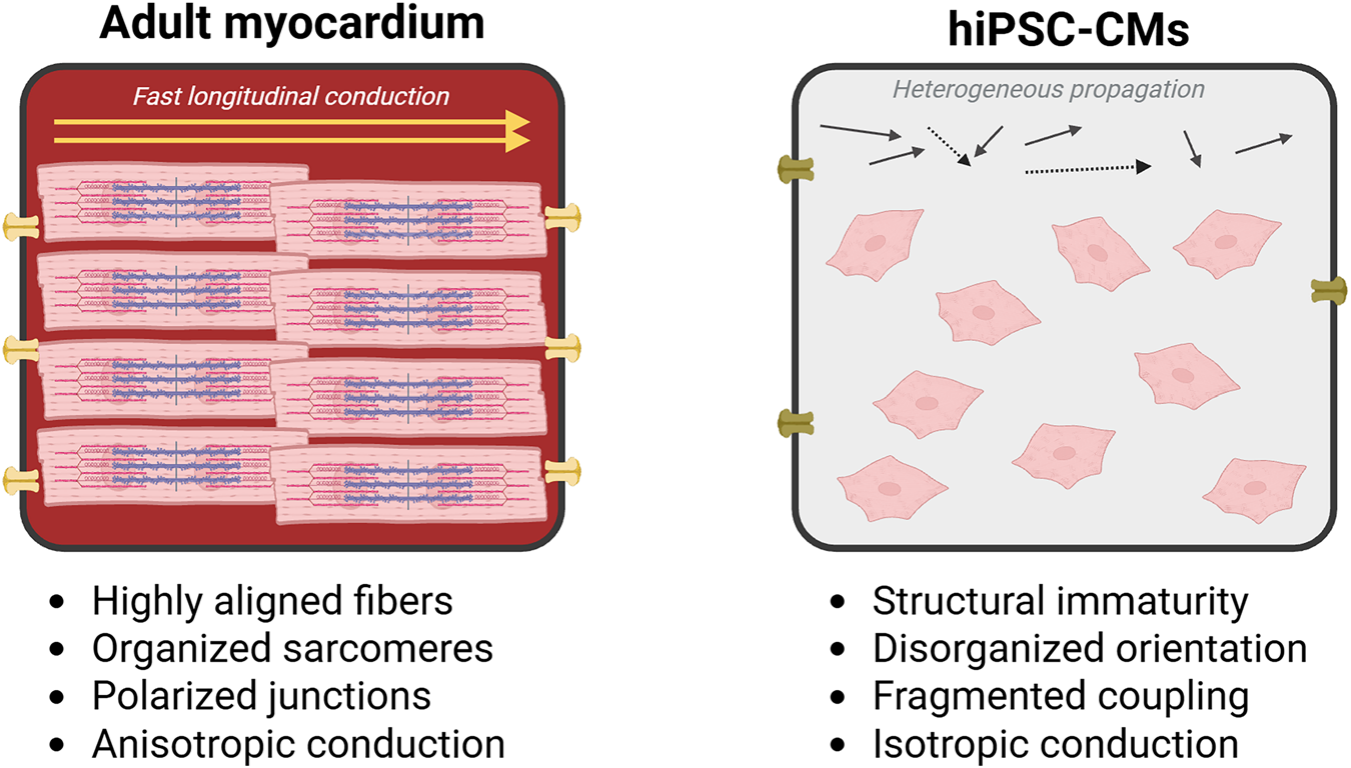
The structural and electrophysiological differences between adult myocardium and immature human induced pluripotent stem-cell-derived cardiomyocytes. Adult ventricular myocardium exhibits highly aligned cardiomyocytes with organized sarcomeres, polarized intercellular junctions and anisotropic electrical conduction that supports rapid longitudinal propagation. In contrast, human induced pluripotent stem cell-derived cardiomyocytes (hiPSC-CMs) are structurally immature and lack coherent alignment, resulting in disorganized orientation, fragmented electrical coupling and heterogeneous, largely isotropic conduction (created in BioRender. Son, Y. (2026) https://BioRender.com/y1ncp32).

A major contributor to these limitations is the structural mismatch between transplanted cardiomyocytes and the highly anisotropic architecture of the native heart (Fig. 1). Adult ventricular myocardium exhibits a well-defined organization in which cardiomyocytes are aligned, supporting directional electrical conduction and efficient force transmission^9,10^. In contrast, hiPSC-CMs have an immature phenotype in structure and function compared with adult heart cells, such as smaller cell size, lack of T tubules and weak contractility^11^. In addition, most hiPSC-CM delivery strategies in stem-cell therapy introduce cells in a disorganized configuration, either as dispersed injections or as grafts with weak or unstable structural cues. In such settings, cardiomyocytes experience abnormal mechanical loading, fragmented cell-to-cell contacts, and non-physiological electrical activation, all of which can compromise their survival, coupling and functional contribution^11^.

This review discusses the perspective that cardiomyocytes alignment is not a secondary feature of successful grafts but rather a foundational requirement for electrical integration and regenerative efficacy. We focus on bioengineering strategies that impose or preserve alignment in hiPSC-CM grafts and examine how structural anisotropy shapes electrophysiology and downstream regenerative outcomes. By reframing cardiomyocyte alignment as a primary design criterion, this review is aimed at highlighting actionable principles for improving the safety and effectiveness of hiPSC-CM based cardiac therapies.

## Why alignment matters: from structure to electrophysiology

Cardiomyocyte alignment refers to the coordinated orientation of cells and their contractile machinery relative to a common axis, recapitulating the anisotropic organization of native myocardium^12^. In hiPSC-CM grafts, alignment can be defined and quantified on multiple structural scales. At the single-cell level, alignment is reflected by elongated cell morphology, oriented nuclei, and parallel sarcomere organization^13^. At the tissue level, alignment describes the degree of coherence in cardiomyocyte orientation across the graft, often quantified using orientation histograms, order parameters or anisotropy indices^14^.

Importantly, alignment is not merely a structural descriptor but a functional determinant of cardiomyocyte behaviour. In aligned hiPSC-CMs, intercellular junctions, including gap junctions and adherens junctions, preferentially localize along the longitudinal cell axis, supporting directional electrical propagation and coordinated excitation^15–17^. This polarized organization facilitates efficient depolarization between neighbouring cells and promotes synchronous activation across the tissue. In contrast, disorganized cardiomyocytes often exhibit fragmented junctional contracts, slowed conduction, conduction block at graft–host interfaces and increased dispersion of activation, all of which compromise electrical coherence and stability^18,19^.

Beyond electrophysiology, alignment also influences mechanical integration and cellular homeostasis^20,21^. Properly aligned cardiomyocytes transmit contractile force more efficiently along defined axes, reducing mechanical inefficiency and abnormal strain distributions^12,22^. Conversely, isotropic or poorly organized grafts experience non-physiological loading, which can disrupt sarcomere integrity, impair excitation–contraction coupling, and exacerbate cellular stress. These effects are particularly relevant for hiPSC-CMs, which are inherently immature and therefore more sensitive to microenvironmental cues.

Together, these observations indicate that cardiomyocyte alignment is a prerequisite for establishing the structural, electrical and mechanical conditions required for healthy myocardial function. Accordingly, restoring or imposing alignment in hiPSC-CM grafts is essential not only for improving electrophysiological integration but also for enabling durable and functionally meaningful cardiac regeneration. In the following sections, we focus on bioengineering strategies that generate reproducible alignment in hiPSC-CM grafts and evaluate how this organization translates into improved electrophysiology and regenerative performance in vivo.

### Conceptual framework and hierarchical definitions of alignment and anisotropy

Cardiomyocyte alignment and anisotropy are frequently used in cardiac bioengineering literature, but they describe related yet distinct phenomena occurring across multiple spatial scales. To clarify terminology used throughout this review, we define key concepts according to a hierarchical framework that distinguishes structural organization at the cellular level from functional behaviour at the tissue level.

#### Single-cell structural alignment

At the most fundamental level, structural alignment refers to the orientation of individual cardiomyocytes relative to a defined axis^23,24^. In hiPSC-CMs, structural alignment is typically assessed through morphological features such as elongated cell shape, nuclear orientation and parallel organization of sarcomeres^25^. These properties reflect how individual cells respond to environmental cues including topographical guidance, mechanical stress or patterned substrates. However, structural alignment at the single-cell level does not necessarily indicate coordinated organization across a multicellular tissue. Individual elongated cells may still be randomly oriented relative to neighbouring cells, resulting in limited functional anisotropy.

#### Tissue order parameters

On the tissue scale, alignment refers to the collective orientation of cardiomyocytes across a population of cells, often described as structural coherence^14,26^. This level of organization is typically quantified using tissue order parameters, which measure the degree of directional uniformity within a cell ensemble^27^. Common approaches include orientation histograms derived from nuclear or sarcomere angles, Fourier-based directional analysis and nematic order parameters, where values approaching 1 indicate highly aligned tissues and values near 0 indicate random orientation^14,21,28–30^. These metrics provide quantitative descriptions of multicellular alignment but remain purely structural measurements. As such, they do not directly reflect the electrical or mechanical performance of the tissue.

#### Functional anisotropy

Functional anisotropy describes direction-dependent electrical propagation within cardiac tissue. In native myocardium, electrical impulses travel more rapidly along the longitudinal axis of cardiomyocytes than across the transverse axis owing to the polarized distribution of gap junctions and coordinated cellular orientation^10,31^. Functional anisotropy therefore emerges from both structural alignment and intercellular coupling. Importantly, morphological alignment alone does not guarantee functional anisotropy^32^. Tissues may appear structurally aligned yet fail to demonstrate directional electrical propagation if intercellular junctions remain immature or if electrical coupling is disrupted.

#### Conduction velocity anisotropy ratio

Functional anisotropy is commonly quantified using the conduction velocity anisotropy ratio, defined as the ratio of longitudinal conduction velocity to transverse conduction velocity^31,33^. In healthy adult myocardium, this ratio typically exceeds 2:1, reflecting efficient anisotropic propagation. Reporting only average conduction velocity may obscure whether physiological anisotropy has been restored, particularly in engineered tissues or transplanted grafts.

#### Hierarchical relationship between alignment and function

Together, these definitions highlight a hierarchical relationship between structural and functional organization:

1. Single-cell alignment — morphology and sarcomere orientation.
2. Multicellular structural coherence — quantified by tissue order parameters.
3. Functional electrical anisotropy — directional conduction behaviour.
4. Organ-level integration — coordinated propagation across graft–host interfaces.

Improvement at one level does not necessarily ensure improvement at higher levels. For example, hiPSC-CMs cultured on patterned substrates may exhibit elongated morphology without demonstrating physiological conduction anisotropy on the tissue scale. Therefore, evaluating alignment-driven therapies requires measurements that span both structural and functional hierarchies.

## Engineering strategies to align transplanted cardiomyocytes

### Topographical guidance cues

Topographical guidance is one of the most direct and reproducible methods for imposing cardiomyocyte alignment. Micro- and nanoscale features such as aligned electrospun fibres, grooves, 3D bio-printed anisotropic scaffolds and patterned hydrogel surfaces provide anisotropic physical features that constrain cytoskeletal organization and promote elongated cardiomyocyte morphology^15,20,27,34–44^. These cues mimic aspects of native extracellular matrix anisotropy and instruct cytoskeletal organization through focal adhesion-mediated signalling.

In hiPSC-CMs, topographical alignment within engineered heart tissues has been shown to enhance structural organization by constraining cell shape and cytoskeletal tension along defined axes, thereby promoting elongated morphology, increased sarcomere length, improved myofibrillar registry and polarized distribution of intercellular junction proteins^15,16,20,34,35,41,45,46^. Unlike randomly seeded cultures, engineered tissues and aligned patches provide persistent physical guidance cues that stabilize cardiomyocyte orientation across multicellular assemblies, enabling alignment to emerge and be maintained on the tissue scale rather than only at the single-cell level.

These architectural constraints translate into functional benefits. Aligned engineered tissues exhibit more uniform calcium transients, improved excitation–contraction coupling and enhanced directional electrical propagation, reflecting reduced cellular heterogeneity and more coherent intercellular coupling^19,20,34,44^. Importantly, when incorporated into cardiac patches, aligned substrates preserve cardiomyocyte orientation at the time of implantation and provide a structural framework that resists the randomization typically induced by intramyocardial injection. By maintaining anisotropy during the critical early engraftment period, engineered tissues and patches increase the likelihood that hiPSC-CMs establish stable electrical and mechanical interactions both within the graft and at the graft–host interface.

However, a key limitation of topographical guidance strategies is the persistence of alignment cues in vivo. Although nanopatterned surfaces and microfabricated platforms consistently promote hiPSC-CM elongation and sarcomere alignment in vitro, these systems often do not address whether anisotropy persists after implantation. Following transplantation, alignment cues may be attenuated by scaffold degradation, foreign-body responses and the non-physiological mechanical environment of scar tissue. Therefore, translationally oriented designs should be evaluated not only by “alignment efficiency” in culture but also by “in vivo stability”, defined as the ability to preserve tissue-scale orientation coherence and directional conduction during early engraftment and remodelling. Table 1 summarizes representative platforms using these two criteria. This highlights the need to design topographical cues that are not only effective in vitro but are also mechanically stable and biologically integrated during the early engraftment period when cardiomyocyte survival and coupling are established.

**Table 1:** Comparison of alignment-enabling platforms by in vitro alignment efficiency and in vivo stability.

Platform	Example cue type	Alignment efficiency	In vivo stability	Translational notes	Refs.
Nano-/micro- patterned surfaces	2D topography	High (single-cell)	Low–moderate (often not implanted)	Excellent for mechanistic studies; limited direct translational maintenance unless transferred into implantable format	^19,20,35,36,39^
Aligned electrospun scaffolds	3D/2.5D topography	High (cell + tissue coherence)	Moderate (depends on degradation/remodelling)	Better implantability; must balance fibre persistence with integration and immune response	^15,16,38,40–42,46^
3D bioprinted anisotropic scaffolds	Architecture + topography	Moderate–High	Uncertain–moderate	Potential to encode spatial anisotropy; in vivo validation still limited	^27,43,44^
Engineered heart tissues/aligned bundles	Architectural alignment	High	Moderate–high	Alignment maintained by tissue architecture; scalability and vascularization are main limits	^34,37,45^

### Mechanical anisotropy and conditioning

Mechanical cues play a central role in regulating cardiomyocyte organization and maturation. In the native myocardium, cardiomyocytes experience cyclic stretching and directional mechanical loading, which contribute to sarcomere organization and contractile coordination. Engineered cardiac tissues that incorporate mechanical conditioning, such as anisotropic stiffness, pre-strained substrates, cyclic mechanical stretch and directionally constrained scaffolds, can promote hiPSC-CM elongation, improve sarcomere maturation and reinforce directional organization by promoting preferential cytoskeletal tension along defined axes^21,47–50^.

**Fig. 2:**
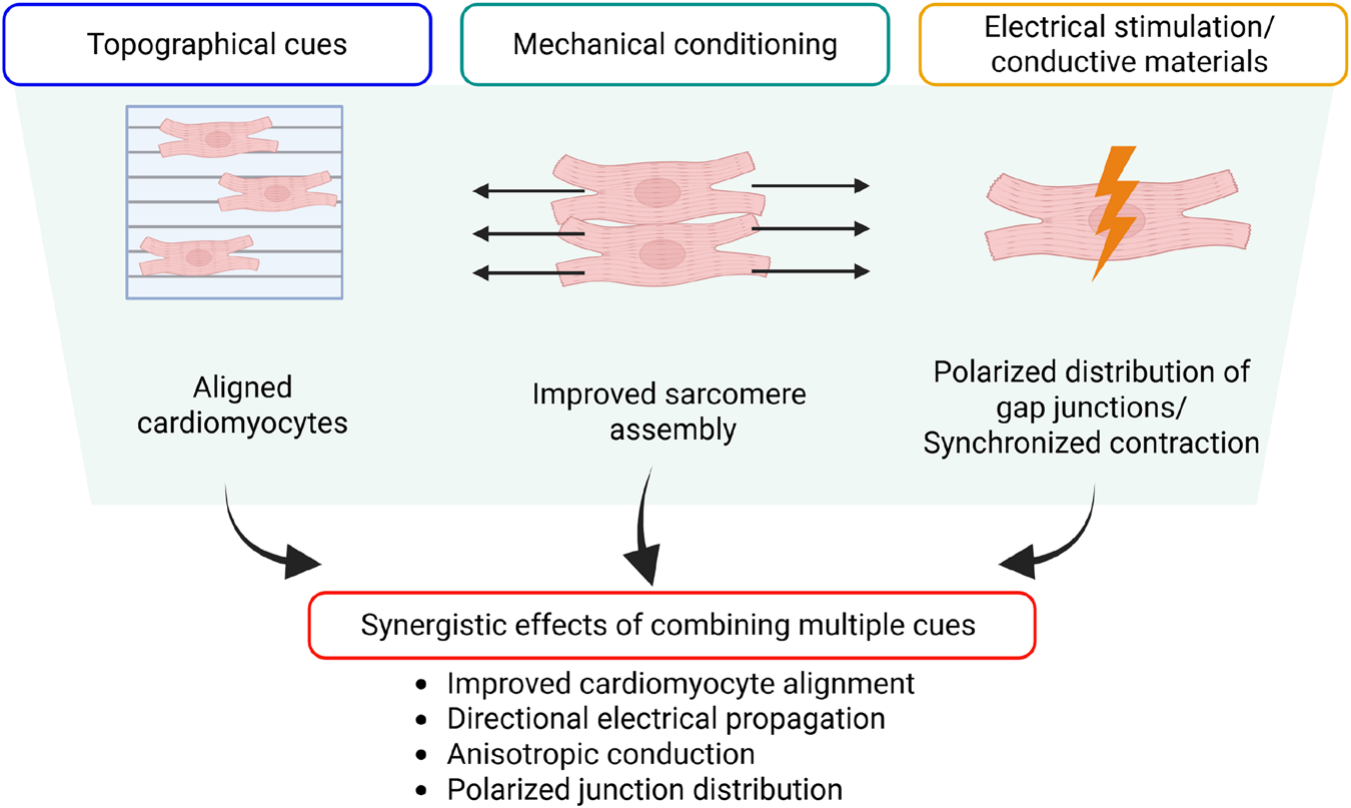
Synergistic engineering cues promote polarized cardiomyocyte organization. Individual bioengineering cues including topographical guidance, mechanical stimulation and electrical conditioning each influence specific aspects of cardiomyocyte structure and function. When combined, these cues can reinforce one another to promote elongated morphology, sarcomere alignment and polarized distribution of intercellular junctions. This coordinated structural organization supports anisotropic electrical propagation and improved electromechanical integration (created in BioRender. Son, Y. (2026) https://BioRender.com/y1ncp32).

Unlike purely topographical cues, mechanical anisotropy directly influences mechanotransduction pathways that regulate sarcomere assembly and junction formation^12,51^. Engineering constructs with stiffness values matched to healthy or peri-infarct myocardium further supports alignment by providing physiological resistance to contraction^52^. When combined with mechanical loading, these systems can reinforce directional organization and improve contractile coordination, effects that are associated with enhanced maturation of hiPSC-CM^53,54^. From an electrophysiological perspective, mechanically aligned tissues exhibit more coherent activation patterns, likely due to improved cell–cell coupling and reduced mechanical heterogeneity that otherwise disrupts conduction^51,54^. Despite these advantages, mechanical conditioning strategies are often applied during in vitro culture and may be difficult to replicate after implantation. How well mechanically induced alignment persists after transplantation remains an open question, particularly in the dynamic and inflammatory environment of the infarcted heart. As a result, mechanical strategies may be more effective when combined with architectural scaffolds that help to preserve tissue-level organization during transplantation, rather than relying solely on preconditioning (Fig. 2).

### Electrical conditioning and conductive materials

Electrical activity is both a consequence and a driver of cardiomyocyte organization^55–57^. In the native myocardium, electrical activation occurs through highly organized conduction pathways that promote synchronized contraction and directional propagation. Replicating aspects of this electrical environment in vitro has been widely explored as a strategy to enhance functional maturation of hiPSC-CMs^58^. Electrical pacing of hiPSC-CM constructs during in vitro culture can synchronize excitation, improve calcium handling and promote the maturation of intercellular junctions such as gap junctions^53,59,60^. In engineered cardiac tissues, electrical conditioning has been shown to enhance conduction velocity and reduce activation heterogeneity, thereby improving the functional coherence of multicellular constructs. These effects primarily influence electrophysiological maturation rather than directly imposing structural orientation.

In parallel, conductive biomaterials have been incorporated into cardiac patches and hydrogels to enhance electrical communication within grafts and across graft–host interfaces^61–63^. Materials incorporating conductive components such as carbon nanotubes and gold nanorods can facilitate electrical signal propagation between cardiomyocytes^55,64–66^. By reducing intercellular resistance across the tissue, these materials may promote more coordinated activation patterns and enhance functional integration of engineered grafts. However, conductivity alone does not recreate the anisotropic conduction of native myocardium. Although improved electrical coupling can enhance synchronized activation, directional electrical propagation still depends on the underlying structural organization of cardiomyocytes. In addition, the long-term biocompatibility and remodelling of some conductive materials remain areas of ongoing investigation, particularly in the context of in vivo implantation.

Consequently, electrical conditioning and conductive materials may be most effective when combined with strategies that directly impose structural alignment (Fig. 2). In such systems, hiPSC-CMs are guided to align structurally while also experiencing coordinated electrical activation, enabling more physiological conduction patterns. This synergy underscores the principle that electrical enhancement should complement, rather than replace, architectural control in aligned cardiac grafts.

### Architectural delivery formats

Beyond microscale cues that guide hiPSC-CM orientation, the architectural format in which engineered tissues are delivered can also influence the degree to which alignment is preserved during transplantation. Delivery strategies, such as engineered heart tissues, aligned patches and bundled cardiomyocyte constructs, organize cells into pre-formed multicellular assemblies prior to implantation. In these formats, cardiomyocyte orientation is established during tissue fabrication and may therefore provide greater control over cellular orientation than intramyocardial injection^47,52,67^.

Epicardial delivery of aligned constructs further allows orientation to be matched to underlying myocardial fibre direction, potentially improving graft–host continuity^47^. In contrast, injected cells are subjected to random mechanical forces and lack structural constraints, often resulting in spherical clusters with poor internal organization. Although architectural grafts are more surgically invasive, they offer a clear advantage in maintaining anisotropy during the critical early post-transplantation period.

However, architectural approaches also present important translational considerations. Many engineered constructs generate uniaxial alignment, whereas the native ventricular myocardium exhibits layered fibre orientations that rotate across the myocardial wall thickness^68,69^. Consequently, a single aligned patch may restore local anisotropy without fully reproducing the three-dimensional architecture required for physiological ventricular contraction. In addition, the optimal orientation of implanted tissues relative to the host myocardium can be difficult to determine, particularly in chronically scarred ventricles where the native anisotropic structure has been disrupted.

These challenges suggest that architectural delivery formats should be considered as one component of a broader strategy to restore myocardial anisotropy. Emerging approaches, including multilayered constructs, spatially patterned scaffolds and adaptive tissue architectures, are aimed at more closely replicating the complex structural organization of native myocardium. Such strategies may ultimately enable engineered grafts to integrate more effectively with host tissue while preserving the anisotropic properties necessary for coordinated cardiac function. Because these strategies target complementary aspects of cardiomyocyte organization, combining structural, electrical and mechanical cues may provide synergistic benefits for promoting alignment and functional maturation. These interactions are summarized schematically in Fig. 2, which illustrates how multiple bioengineering cues can cooperatively drive polarized intercellular junction distribution and anisotropic conduction in engineered cardiac tissues.

## Discussion

Despite growing recognition of the importance of cardiomyocyte alignment, several challenges limit its consistent implementation and evaluation. First, the metrics used to quantify alignment remain highly inconsistent across studies. Alignment has been assessed using a wide range of approaches, including qualitative image inspection, nuclear orientation angles, sarcomere orientation distributions, Fourier-based analysis and tissue-level order parameters^14,21,28–30^. Although each metric captures a valid aspect of structural organization, their inconsistent application and reporting make direct comparison between studies difficult and obscure the relationship between alignment and functional outcomes. In some cases, alignment is inferred from representative images alone, without quantitative thresholds or statistical analysis, limiting interpretability. This variability contrasts with the more standardized framework commonly applied to electrophysiological assessment, where parameters such as conduction velocity, activation maps and anisotropy ratios are increasingly reported using established methodologies. As a result, electrophysiological improvements are often evaluated with greater rigour than the structural alignment that is presumed to underlie them. Without harmonized alignment metrics, it remains challenging to determine whether functional differences arise from true differences in tissue organization or from confounding factors such as cell density, maturation state or graft size.

Second, electrophysiological assessments often rely on global measures, such as average conduction velocity or ejection fraction, without explicitly testing conduction anisotropy^70,71^. This is particularly limiting after cell transplantation, where grafts introduce spatially heterogeneous tissue with distinct structural and electrical properties. Without directional measurements, improvements in average conduction may mask localized conduction slowing, block, or graft–host discontinuities, thereby obscuring the true contribution of cardiomyocyte alignment to functional and electrophysiological outcomes^4,72,73^.

These considerations are closely related to an important safety consideration in cardiac cell therapy, namely ventricular arrhythmias following hiPSC-CM transplantation. Preclinical large animal studies and the first human trial have reported episodes of ventricular tachycardia after transplantation of hiPSC-CMs, highlighting the importance of understanding how graft structure may influence the electrophysiological substrate^74–77^. However, these events are often transient and occur predominantly during the early post-engraftment period, and the overall arrhythmogenic risk of hiPSC-CMs appears lower than for other cell types owing to their capacity for electrical coupling and progressive maturation in vivo^75,78^. Structural disorganization within transplanted cardiomyocyte populations may nonetheless contribute to a transient pro-arrhythmic substrate through several mechanisms. Randomly oriented cardiomyocytes can generate spatially heterogeneous anisotropy, leading to local variations in conduction velocity that may promote conduction slowing or block. Such heterogeneity can increase the likelihood of abnormal electrical loops, especially when combined with discontinuities in electrical propagation. In addition, abrupt changes in fibre orientation or electrical coupling at the graft–host interface may create alignment mismatches that further disrupt wavefront propagation, especially in scarred myocardium where native anisotropy is already impaired^79^. Immature electrophysiological properties of hiPSC-CMs, including automaticity and prolonged action potential duration, may also contribute to triggered activity and ectopic rhythms^80^. Although alignment alone is unlikely to eliminate these risks, restoring controlled anisotropy may promote more uniform and predictable wavefront propagation, thereby reducing conduction dispersion and potentially improving the electrical stability of engineered grafts.

From a bioengineering perspective, several design principles emerge. Alignment cues must be sufficiently strong to persist during the early engraftment phase, when cardiomyocyte survival and coupling are established. Structural anisotropy should be integrated with mechanical compliance and electrical support, rather than applied in isolation. In addition, the orientation of the graft relative to host myocardial fibres is probably as important as alignment within the graft itself, underscoring the need to engineer continuity across the graft–host interface. In practice, determining the optimal orientation of implanted tissues remains challenging, particularly in infarcted myocardium where native fibre architecture may be disrupted. Emerging imaging approaches such as diffusion tensor (DT)-MRI and other fibre-mapping techniques may help to estimate local myocardial orientation and guide graft placement^81^. Alternatively, engineering strategies that incorporate multilayered or rotationally patterned constructs may reduce the need for precise alignment with a single fibre direction by accommodating the layered anisotropy of the ventricular wall^68,69^.

Translational challenges also remain. Aligned patches and engineered tissues are more complex to manufacture and implant than injectable cell suspensions. Graft thickness, vascularization and immune compatibility further constrain scalability^82,83^. Addressing these issues will require the development of minimally invasive, alignment-preserving delivery platforms that balance structural control with clinical feasibility. Finally, alignment-driven benefits must be separated from confounding factors such as cell dose, maturation state and retention. Standardized reporting of alignment metrics and anisotropy-aware electrophysiological endpoints will be essential for advancing the field.

## Conclusions and future directions

Cardiomyocyte alignment is a fundamental determinant of electrophysiological integration and regenerative performance in hiPSC-CM-based cardiac therapies. Rather than serving as a secondary or cosmetic feature, alignment shapes survival, electrical coupling and functional contribution by restoring key aspects of myocardial anisotropy. Bioengineering strategies that impose or preserve alignment have demonstrated consistent improvements in structural organization and directional conduction, highlighting alignment as an actionable design parameter. Future efforts should prioritize three areas. First, graft–host interface engineering must ensure continuity of fibre orientation to prevent conduction discontinuities^44^. Second, electrophysiological evaluation should incorporate anisotropy-aware metrics to accurately assess safety and function. Third, scalable and clinically feasible delivery formats that maintain alignment during and after implantation are needed to translate these advances beyond experimental settings. By centring cardiomyocyte alignment as a primary design criterion, the field can move toward cardiac cell therapies that not only generate new myocardium but also restore the structural and functional principles that underpin a healthy heart.
